# Differentiation Between Benign and Metastatic Breast Lymph Nodes Using Apparent Diffusion Coefficients

**DOI:** 10.3389/fonc.2022.795265

**Published:** 2022-02-23

**Authors:** Reza Fardanesh, Sunitha B. Thakur, Varadan Sevilimedu, Joao V. Horvat, Roberto Lo Gullo, Jeffrey S. Reiner, Sarah Eskreis-Winkler, Nikita Thakur, Katja Pinker

**Affiliations:** ^1^Department of Radiology, Memorial Sloan Kettering Cancer Center, New York, NY, United States; ^2^Department of Medical Physics, Memorial Sloan Kettering Cancer Center, New York, NY, United States; ^3^Department of Epidemiology and Biostatistics, Memorial Sloan Kettering Cancer Center, New York, NY, United States; ^4^Touro College of Osteopathic Medicine, Middletown, NY, United States

**Keywords:** breast cancer, prognostic factors, lymph nodes, diffusion-weighted imaging, apparent diffusion coefficient, MRI

## Abstract

The aim of this study was to determine the range of apparent diffusion coefficient (ADC) values for benign axillary lymph nodes in contrast to malignant axillary lymph nodes, and to define the optimal ADC thresholds for three different ADC parameters (minimum, maximum, and mean ADC) in differentiating between benign and malignant lymph nodes. This retrospective study included consecutive patients who underwent breast MRI from January 2017–December 2020. Two-year follow-up breast imaging or histopathology served as the reference standard for axillary lymph node status. Area under the receiver operating characteristic curve (AUC) values for minimum, maximum, and mean ADC (min ADC, max ADC, and mean ADC) for benign *vs* malignant axillary lymph nodes were determined using the Wilcoxon rank sum test, and optimal ADC thresholds were determined using Youden’s Index. The final study sample consisted of 217 patients (100% female, median age of 52 years (range, 22–81), 110 with benign axillary lymph nodes and 107 with malignant axillary lymph nodes. For benign axillary lymph nodes, ADC values (×10^−3^ mm^2^/s) ranged from 0.522–2.712 for mean ADC, 0.774–3.382 for max ADC, and 0.071–2.409 for min ADC; for malignant axillary lymph nodes, ADC values (×10^−3^ mm^2^/s) ranged from 0.796–1.080 for mean ADC, 1.168–1.592 for max ADC, and 0.351–0.688 for min ADC for malignant axillary lymph nodes. While there was a statistically difference in all ADC parameters (p<0.001) between benign and malignant axillary lymph nodes, boxplots illustrate overlaps in ADC values, with the least overlap occurring with mean ADC, suggesting that this is the most useful ADC parameter for differentiating between benign and malignant axillary lymph nodes. The mean ADC threshold that resulted in the highest diagnostic accuracy for differentiating between benign and malignant lymph nodes was 1.004×10^−3^ mm^2^/s, yielding an accuracy of 75%, sensitivity of 71%, specificity of 79%, positive predictive value of 77%, and negative predictive value of 74%. This mean ADC threshold is lower than the European Society of Breast Imaging (EUSOBI) mean ADC threshold of 1.300×10^−3^ mm^2^/s, therefore suggesting that the EUSOBI threshold which was recently recommended for breast tumors should not be extrapolated to evaluate the axillary lymph nodes.

## Introduction

Unspecific axillary lymphadenopathy is often encountered in breast imaging. It may be caused by various benign conditions (1, 2) or more recently after COVID-19 vaccinations (3, 4); therefore, patients with a personal history or concurrent diagnosis of breast cancer in particular can pose a diagnostic dilemma. In patients with breast cancer, axillary lymph node status is an important prognostic factor (5) and one of the strongest predictors of late distant recurrence (6). Sentinel lymph node biopsy is a standard procedure in early-stage breast cancer patients with clinically negative axillary lymph nodes (7), with a reported sensitivity of 58%–72% (8–10) and accuracy of 75% (11). However, while it is a minimally invasive procedure, it is associated with several morbidities, e.g., lymphedema (8.2%) (12), seroma (19.5%), localized swelling, pain and paresthesia, infectious neuropathy, decreased arm strength, and shoulder stiffness (13).

In both scenarios of lymphadenopathy with and without a personal history of breast cancer, the use of a non-invasive imaging technique for the accurate assessment of axillary nodal status is thus desirable. On magnetic resonance imaging (MRI), differentiating between malignant and benign axillary lymph nodes is challenging when the evaluation is made solely on the basis of morphological criteria (14–16). Indeed, prior studies evaluating the axilla with MRI have reported a mean accuracy of only 75% (range, 71%–85%) in predicting axillary metastasis (17–19).

The addition of functional imaging parameters such as diffusion-weighted imaging (DWI) to dynamic contrast-enhanced MRI, i.e., in a multiparametric MRI framework, has been shown to improve diagnostic accuracy for evaluating breast tumors (20–23). DWI using apparent diffusion coefficient (ADC) mapping has a reported sensitivity of up to 96% and specificity of up to 100% for breast cancer detection (24, 25). While the primary use of DWI is to improve the differentiation between benign and malignant lesions to prevent unnecessary breast biopsies (26–29), in recent years, DWI has also shown promise in axillary lymph node mapping (15, 30, 31).

Recently, the European Society of Breast Imaging (EUSOBI) provided evidenced-based levels of diffusion restriction for breast tumors, aiming towards the assessment of breast lesions using DWI in an objective way (32). In daily clinical practice, benign axillary lymph nodes can nevertheless present with a wide range of ADC mean values, some even falling well below the lower limit of the range prescribed by EUSOBI for benign tumors. While several studies have shown that ADC values are promising to differentiate between benign and malignant lymph nodes in breast cancer patients, the possible range of ADC values for benign axillary lymph nodes and its associated possible clinical indications has yet to be delineated. In addition, it remains unknown how the recently proposed levels of diffusion restriction for breast tumors would perform in axillary lymph nodes, i.e., if they can be extrapolated to the axilla.

Therefore, the aim of this study was to determine the range of ADC values for benign axillary lymph nodes in contrast to malignant lymph nodes, and to define the optimal ADC threshold for three different ADC parameters (minimum, maximum, and mean ADC) in differentiating between benign and malignant lymph nodes. Secondarily, to determine if the mean ADC threshold recently prescribed by EUSOBI for the differentiation of benign and malignant breast tumors can be extrapolated to evaluate axillary lymph nodes, the study aimed to compare the performance of mean ADC using the optimal mean ADC threshold as determined in this study as opposed to the threshold prescribed by EUSOBI.

## Materials and Methods

### Patients

This retrospective study was approved by the institutional review board at Memorial Sloan Kettering Cancer Center and the requirement for informed consent was waived. All study procedures were conducted according to the Declaration of Helsinki. Two separate groups of consecutive patients who underwent breast MRI at a tertiary care center from January 2017–December 2020 were identified. Group one (patients with benign axillary lymph nodes) were patients with a Breast Imaging and Reporting and Data System (BI-RADS) score of 1 or 2 on MRI and subsequent negative two-year follow-up breast MRI. Of 268 patients who fulfilled these criteria, 158 patients were excluded due to either DWI sequences not performed or no measurable axillary lymph nodes in the field of view of DWI. Group two (patients with metastatic axillary lymph nodes, i.e., malignant lymph nodes) were patients with a BI-RADS score of 6 on MRI with a subsequent biopsy that showed morphologically abnormal adenopathy. Of 317 patients who fulfilled these criteria, 210 patients were excluded due to either DWI sequences not performed, axillary lymph nodes not in the field of view of DWI, or only post-neoadjuvant MRI exam available. The final study sample consisted of 217 patients, 110 who had benign lymph nodes and 107 who had malignant lymph nodes.

### Magnetic Resonance Imaging Protocol

All MRI examinations were performed using a 3 Tesla system (Discovery MR750; GE Healthcare, Chicago, IL) with a dedicated 16-channel phased-array breast coil (Vanguard, Sentinelle Medical, Toronto, Canada), with patients in the prone position. A standard multiparametric breast protocol was performed including axial T2-weighted imaging with and without fat saturation, DWI with ADC mapping, and dynamic contrast-enhanced imaging after an injection of a standard dose of contrast agent (0.1 mmol/kg bodyweight).

Axial DWI was performed using single-shot spin echo sequence with echo-planar imaging readouts, with b-values of 0 and 800 s/mm^2^. Parameters were as follows: TR, 6000 ms; TE, minimum, flip angle, 90°; acquisition matrix, 192 × 192; reconstructed matrix, 256 × 256; FOV, 28–38 cm; slice thickness, 3.9 mm; NEX, 3; slice gap, 0–1 mm; fat suppression, special; parallel imaging, ASSET; acquisition time, 3-4 minutes. Dual shim volumes were placed over both breasts to optimize the B_0_ homogeneity.

### Image Analysis

All MR images were reviewed by one radiologist with subspecialty training in breast MRI interpretation. Lymph nodes were identified on the ADC map by using conventional MR imaging information as a reference. Measurements were performed by placing a region of interest (ROI) of 0.5 mm diameter on lesions. 2D regions of interest (ROIs) measuring at least 5 mm were drawn manually on ADC maps within the solid portion on the largest section of lymph node. ADC values were measured three times in three different evaluation sessions and averaged as means ± standard deviations.

### Statistical Analysis

Descriptive characteristics were summarized using medians and interquartile ranges (IQR).

Minimum, maximum, and mean ADC (min ADC, max ADC, and mean ADC) were compared between benign and malignant lymph nodes using the Wilcoxon rank sum test. To compare the accuracy of these three ADC parameters in discriminating between benign and malignant axillary lymph nodes, area under the receiver operating characteristic curve (AUC) with 95% confidence intervals were compared using DeLong’s test for correlated receiving operating characteristic curves (33), with Bonferroni correction for multiple comparisons (α* = 0.016).

Thresholds (optimal cut-off points) for discriminating between benign and malignant axillary lymph nodes using the three parameters were estimated using Youden’s Index, and sensitivity, specificity, positive predictive value (PPV), negative predictive value (NPV), and accuracy for each parameter were determined at the corresponding optimal thresholds. Sensitivity and specificity of the mean ADC parameter using the determined optimal threshold for axillary lymph nodes *vs.* the EUSOBI ADC threshold for breast tumors were compared using McNemar’s test with continuity correction. All statistical analysis was done using R 3.6.3.

## Results

### Patient Characteristics

The study sample consisted of 217 patients (100% female) with a median age of 52 years (range, 22–81). All patients with benign axillary lymph nodes had no known prior history of breast cancer.

### Range of ADC Values of Benign and Malignant Axillary Lymph Nodes

**Figure 1:**
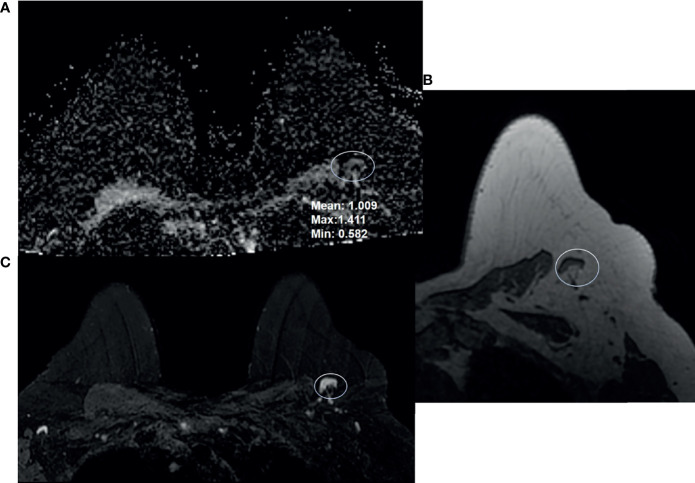
68-year-old woman presented for high-risk screening breast MRI exam. She had a family history of cancer, and was BRCA1 and ATM positive. Breast MRI shows a benign appearing left axillary level 1 lymph node: **(A)** ADC, **(B)** T1-weighted non-fat saturated, and **(C)** T2-weighted fat saturated axial sequences.

**Figure 2:**
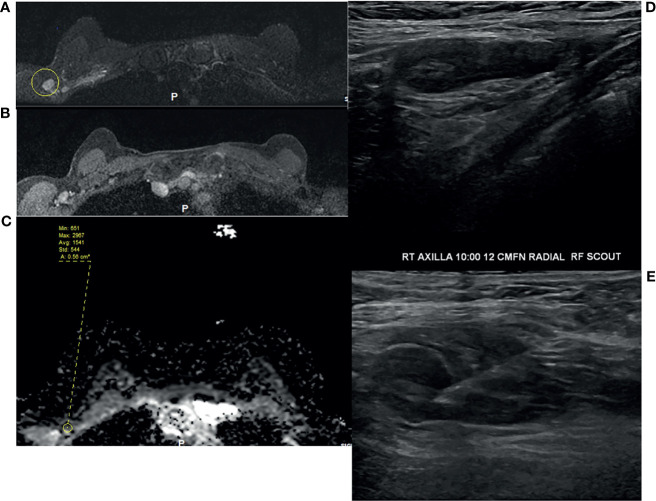
49-year-old woman underwent routine screening breast MRI exam. She received a dose of the COVID vaccine in the right arm a few months prior to her breast MRI. Enlarged right axillary lymph nodes were identified on breast MRI. **(A)** T2-weighted fat saturated image, **(B)** T1-weighted fat saturated post-contrast image, and **(C)** ADC. She subsequently underwent diagnostic ultrasound and ultrasound-guided fine needle aspiration **(D, E)**, which yielded benign results.

In patients with benign axillary lymph nodes, ADC values (× 10^−3^ mm^2^/s) ranged from 0.522–2.712 for mean ADC, 0.774–3.382 for max ADC, and 0.071–2.409 for min ADC. The median values (× 10^−3^ mm^2^/s) of mean ADC, max ADC, and min ADC in these patients were 1.214 (median IQR from 1.022–1.469), 1.674 (median IQR 1.370-2.122), and 0.764 (median IQR 0.535-0.981), respectively (**Table 1**) (**Figures 1**, **2**).

**Table 1:** Comparison of ADC parameters between benign and malignant lymph nodes.

Characteristic	Overall	Benign	Malignant	p-value
(median value)	(n = 217)	(n = 110)	(n = 107)	
ADC mean (× 10^−3^ mm^2^/s)	1.033 (0.911, 1.254)	1.214 (1.022, 1.469)	0.942 (0.796, 1.080)	1.506 × 10^−14^
ADC max (× 10^−3^ mm^2^/s)	1.486 (1253, 1.772)	1.674 (1.370, 2.122)	1.392 (1.168, 1.592)	1.284 × 10^−7^
ADC min (× 10^−3^ mm^2^/s)	0.642 (0.462, 0.841)	0.764 (0.535, 0.981)	0.540 (0.351, 0.688)	2.197 × 10^−8^

**Figure 3:**
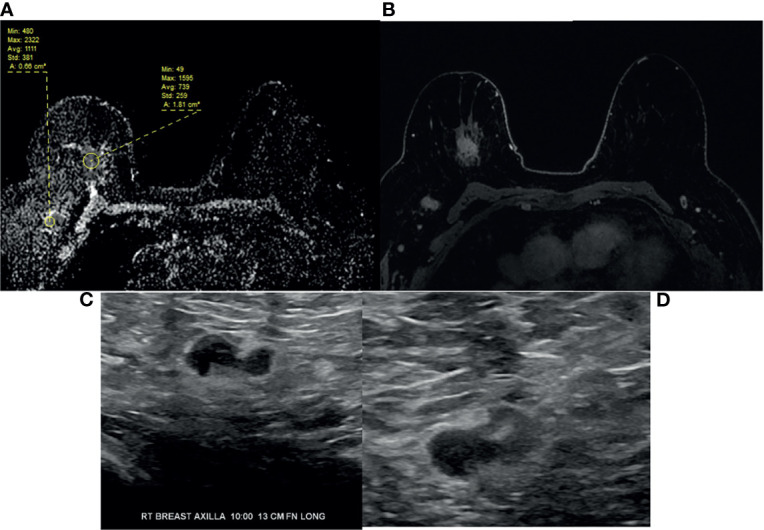
64-year-old woman with right breast 12:00 axis triple negative invasive ductal carcinoma and right axillary lymphadenopathy as seen on **(A)** ADC, **(B)** T1-weighted fat saturated post-contrast image. **(C)** Targeted ultrasound shows borderline cortical thickening of the right axillary lymph node. **(D)** Ultrasound-guided fine needle aspiration confirmed metastatic adenopathy. Note the difference in ADC values between primary breast and right axillary adenopathy, e.g., mean ADC 0.739 versus 1.111.

In patients with malignant axillary lymph nodes, ADC values (× 10^−3^ mm^2^/s) ranged from 0.432–1.570 for mean ADC, 0.478–2.203 for max ADC, and 0.008–1.251 for min ADC. The median values (× 10^−3^ mm^2^/s) of mean ADC, max ADC, and min ADC in these patients were 0.942 (median IQR 0.796–1.080), 1.392 (median IQR 1.168–1.592), and 0.540 (median IQR 0.351–0.688), respectively (**Table 1**) (**Figure 3**).

**Figure 4:**
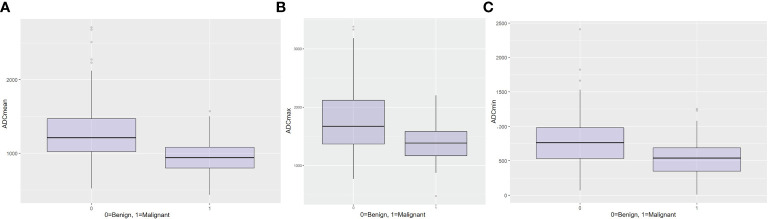
Boxplots for **(A)** mean ADC, **(B)** maximum ADC, and **(C)** minimum ADC for the differentiation between benign and malignant axillary lymph nodes.

While there was a statistically difference in all ADC parameters (p < 0.001) between benign and malignant axillary lymph nodes (**Table 1**), boxplots for mean (**Figure 4A**), max (**Figure 4B**), and min ADC (**Figure 4C**) illustrate that there is an overlap of benign and malignant nodes which is the least for ADC mean, indicating that this is the most useful metric.

### ADC Thresholds for Differentiating Between Benign and Malignant Axillary Lymph Nodes

**Figure 5:**
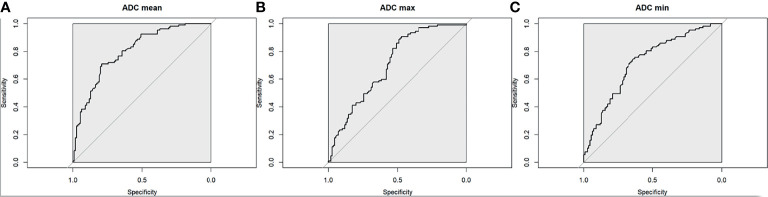
Receiver operating characteristic curve for **(A)** mean ADC, **(B)** maximum ADC, and **(C)** minimum ADC for the differentiation between benign and malignant axillary lymph nodes.

The optimal mean ADC threshold for differentiating between benign and malignant axillary lymph nodes was 1.004 × 10^−3^mm^2^/s, yielding an accuracy of 75% (95% CI 0.688, 0.807), sensitivity of 71% (95% CI 0.615, 0.794), specificity of 79% (95% CI 0.703, 0.863), PPV of 77% (95% CI 0.672, 0.847), and NPV of 74% (95% CI 0.648, 0.814) (**Figure 5A**).

The optimal max ADC threshold for differentiating between malignant and benign axillary lymph nodes was 1.740 × 10^−3^ mm^2^/s, yielding an accuracy of 69% (95% CI 0.62, 0.748), sensitivity of 91% (95% CI 0.835, 0.954), specificity of 47% (95% CI 0.377, 0.57), PPV of 63% (95% CI 0.545, 0.702), and NPV of 84% (95% CI 0.723, 0.92) (**Figure 5B**).

The optimal min ADC threshold for differentiating between malignant and benign axillary lymph nodes was 0.692 × 10^−3^ mm^2^/s, yielding an accuracy of 69% (95% CI 0.625, 0.752), sensitivity of 76% (95% CI 0.665, 0.835), specificity of 63% (95% CI 0.53, 0.718), PPV of 66% (95% CI 0.573, 0.747), and NPV of 73% (95% CI 0.625, 0.813) (**Figure 5C**).

### Comparison of the Determined Optimal Mean ADC Threshold *vs.* EUSOBI Mean ADC Threshold

Compared with the determined optimal mean ADC threshold of 1.004 × 10^−3^mm^2^/s, when the EUSOBI mean ADC threshold of 1.300 x 10^−3^ mm^2^/s was applied to axillary lymph nodes, it had lower discriminative power to differentiate between benign and malignant axillary lymph nodes, yielding an accuracy of 66% (95% CI 0.60, 0.73), with a sensitivity of 94% (95% CI 0.88, 0.98), specificity of 39% (95% CI 0.30, 0.49), PPV of 60% (95% CI 0.52, 0.68), and NPV of 88% (95% CI 0.75, 0.95). While the EUSOBI mean ADC threshold had a significantly higher sensitivity than the optimal mean ADC threshold (p ≤ 0.001), it had a significantly lower specificity (p < 0.001). Specifically, there were 6 false-negative findings using the EUSOBI mean ADC threshold versus 31 false-negative findings using the optimal mean ADC threshold, while there were 67 false-positive findings using the EUSOBI mean ADC threshold versus 23 false-positive findings using the optimal mean ADC threshold. An ADC mean threshold of 1.004 × 10^−3^ would have obviated 66% of recommendations for biopsies in benign lymph nodes.

We further validated our findings by performance of an analysis based on an internal validation cohort obtained by random sampling of 50% of the original cohort, which yielded similar results: (a) the minimum, maximum, and mean ADC values were significantly different between benign and malignant nodes, (b) the ROC obtained by using mean ADC values was significantly better than those obtained by using minimum and maximum values (p = 0.04664 and 0.00336, respectively), (c) sensitivity was better with the EUSOBI threshold (*vs* the proposed ADC mean threshold) (McNemar test p = 0.004427) while specificity was better with the proposed ADC mean threshold of 1.004 × 10^−3^ (McNemar’s test p = 0.0001) and (d) mean ADC values provided the least overlap between benign and malignant nodes.

## Discussion

In this study, while significant differences were observed when comparing the median values all ADC parameters (mean ADC, max ADC, and min ADC) between benign and malignant axillary lymph nodes, results show that there is a significant overlap of ADC values of benign and malignant nodes. The least overlap in ADC values occurred with mean ADC, suggesting that this is the most useful ADC parameter for differentiating between benign and malignant axillary lymph nodes. The mean ADC threshold that resulted in the highest diagnostic accuracy for differentiating between benign and malignant lymph nodes was 1.004 × 10^−3^ mm^2^/s, which is lower than the EUSOBI mean ADC threshold of 1.300 x 10^−3^ mm^2^/s which was recently recommended for breast tumors but not for axillary lymph nodes per se.

The median values of mean ADC, max ADC, and min ADC were significantly lower for malignant *vs.* benign axillary lymph nodes, in agreement with the findings from a meta-analysis of ten studies, where the mean ADC value of metastatic lymph nodes was also significantly lower than that of benign axillary lymph nodes (34). Our data indicate, however, that while these differences were significant (p < 0.001), the range of possible ADC values for benign axillary lymph nodes was wide, overlapping with the range of possible ADC values for malignant axillary lymph nodes both in our study as well as in previous studies in the literature.

Previously published data have shown that malignant nodes can also present with a range of ADC values from 0.666×10^−3^ mm^2^/s to 1.369×10^−3^ mm^2^/s (21, 23–25), with the discrepancies between studies most likely due to differences in nodal tumor burden. Yamaguchi et al. (21) reported a mean ADC value of metastatic axillary lymph nodes ranging from 0.553×10^−3^ mm^2^/s to 1.135×10^−3^ mm^2^/s. Fornasa et al. (30, 35) reported a mean ADC value of 0.878 × 10^−3^ mm^2^/s (range, 0.30–1.20 × 10^−3^ mm^2^/s) in 43 metastatic axillary lymph nodes. Our study found the median value of mean ADC to be 1.214 × 10^−3^ mm^2^/s (range, 0.522–2.712 × 10^−3^ mm^2^/s).

In our study, a similar range was also identified for benign axillary lymph nodes, which had a mean ADC ranging from 0.522–2.712 ×10^−3^ mm^2^/s, max ADC from 1.788–3.382 ×10^−3^ mm^2^/s, and min ADC from 0.71–2.409 ×10^−3^ mm^2^/s. These values overlap with that of malignant axillary lymph nodes not only with those in our study but also with those reported in the literature (36–38). This overlap can present diagnostic challenges, e.g., in patients with a current or past personal history of breast cancer or in the setting of morphologically abnormal yet benign lymph nodes due to conditions such as vaccination. As the degree of overlap was least for mean ADC, this suggests that it would be the most useful ADC parameter for characterizing axillary lymph nodes.

In our study, the optimal mean ADC threshold for differentiating between benign and malignant nodes was 1.004 × 10^−3^ mm^2^/s, which is in line with prior studies investigating axillary lymph nodes in patients with breast cancer (36, 37). For example, Hasanzadeh et al. reported that the optimal mean ADC cut-off value for differentiating between metastatic and non-metastatic axillary lymph nodes was 0.904 × 10^−3^ mm^2^/s, which yielded a higher specificity (88.9%) and accuracy (91.8%) than min ADC or max ADC (39). Yamaguchi et al. (36) reported a sensitivity and specificity of 85% and 81%, respectively, for differentiating metastatic from non-metastatic axillary lymph nodes using a cut-off ADC value of 0.852. Kamitani et al. (37) reported a sensitivity of 53.8%, specificity of 86.9%, PPV of 56.0%, NPV of 85.9%, and accuracy of 79.1% with a mean ADC ≤ 1.05 × 10^−3^ mm^2^/s.

The EUSOBI International Breast DWI working group recently issued a consensus and mission statement that included acquisition parameters for standard breast DWI sequences including specifications of b values, fat saturation, spatial resolution, and repetition and echo times, as well as levels of diffusion restriction/hindrance in the breast based on the published literature on breast DWI to allow the assessment of breast lesions in an objective way (32). The use of ADC values measured at the high b value of 800 s/mm^2^ was recommended, with diffusion levels in lesions classified as follows: very low (ADC = ≤ 0.9 × 10^−3^ mm^2^/sec); low (ADC = 0.9–1.3 × 10^−3^ mm^2^/s); intermediate (ADC = 1.3–1.7 × 10^−3^ mm^2^/s); high (ADC = 1.7–2.1 × 10^−3^ mm^2^/s) and very high (ADC > 2.1 x 10^−3^ mm^2^/s) (32). Lesions with very low and ADC values are considered suspicious for malignancy and biopsy is recommended for these lesions. However, it was unclear how the recently proposed levels of diffusion restriction for breast tumor perform in axillary lymph nodes.

In our study, based on ROC curve analysis, the optimal mean ADC threshold for differentiating between malignant and benign lymph nodes was 1.004 × 10^−3^ mm^2^/s, resulting in a diagnostic accuracy of 75%. This threshold is lower the EUSOBI mean ADC that is recommended for breast tumors. When the EUSOBI mean ADC threshold was applied to axillary lymph nodes in our study, the diagnostic accuracy dropped to 66%. Moreover, the specificity also dropped from 79% to 39%. This suggests that the EUSOBI mean ADC threshold for characterizing breast tumors does not equally apply to the characterization of axillary lymph nodes and different thresholds are needed for these entities. However, it has to be noted that the optimal mean ADC threshold of 1.004 × 10^−3^ mm^2^/s yielded a lower sensitivity than the EUSOBI mean ADC; thus, if the threshold of 1.004 × 10^−3^ mm^2^/s is used, recommendations for biopsy versus follow-up will have to be made carefully in consideration of the clinical context.

Another option would be to consciously select a more conservative threshold that decreases specificity and increases sensitivity. It has been shown in breast tumors that the selection of ADC cut-off values to characterize breast tumors can be dependent on the expectations from DWI (40). Higher cut-off values are desirable for increasing sensitivity, whereas lower cut-off values are desirable for increasing specificity. The recent American College of Radiology Imaging Network 6702 trial evaluated the ADC values of undiagnosed breast lesions (BI-RADS 3, 4, or 5) identified at DCE-MRI and proposed an ADC cut-off of 1.68 × 10^−3^ mm^2^/s to improve specificity without affecting sensitivity (41). For the assessment of axillary lymph nodes, currently it seems that the suspicion of malignancy should therefore be interpreted in conjunction with the patient’s history (past or current diagnosis of breast cancer, vaccination status), lymph node morphology (cortical thickness), and if applicable the ADC values of the index cancer.

This study has limitations. It was a single-center study and therefore it was difficult to predict how the thresholds might perform with data acquired using different imaging protocols. Nevertheless, the thresholds were in line with prior studies from different institutions performed in patients with breast cancer. In our study, a single-shot EPI DWI sequence was used, and therefore, our results may not be extrapolated to other DWI sequences. In addition, there are constant improvements in DWI techniques (42, 43) and the use of more advanced techniques may further improve axillary lymph node assessment. In this study, long-term stability of axillary lymph nodes indicated by least two years of negative follow-up MRI was required to establish benign status. Therefore, we did not include datag acquired with the recently implemented advanced high-spatial-resolution multishot multiplexed sensitivity-encoding DWI at our institution, but this will be the focus of a future study.

In conclusion, benign axillary lymph nodes can present with a wide range of ADC values. While there are significant differences in ADC values between benign and malignant axillary lymph nodes, radiologists should be aware of a significant overlap, with mean ADC possibly being the most useful ADC parameter in this context. The mean ADC threshold that provided the highest diagnostic accuracy for differentiation between benign and malignant axillary lymph nodes is lower than the threshold recommended by EUSOBI for breast tumors; hence, the latter threshold should not be extrapolated to the axilla to avoid unnecessary biopsies.

## Data Availability Statement

The datasets generated during and/or analyzed during the current study are available from the corresponding author on reasonable request. Requests to access these datasets should be directed to KP, pinkerdk@mskcc.org.

## Ethics Statement

The studies involving human participants were reviewed and approved by Institutional Review Board, Memorial Sloan Kettering Cancer Center. Written informed consent for participation was not required for this study in accordance with the national legislation and the institutional requirements.

## Author Contributions

KP, RF, ST, and VS contributed to conception and design of the study. RF, JH, RL, JR, ESW, and NT organized the database. VS performed the statistical analysis. RF wrote the first draft of the manuscript. JH, RL, JR, and ESW wrote sections of the manuscript. All authors contributed to revising the manuscript and approved the submitted version of the manuscript.

## Funding

This research was funded in part through the NIH/NCI Cancer Center Support Grant P30 CA008748 and the NIH/NCI UG3 CA239861 grant.

## Conflict of Interest

KP received payment for activities not related to the present article including lectures and service on speakers bureaus and for travel/accommodations/meeting expenses unrelated to activities listed from the European Society of Breast Imaging (MRI educational course, annual scientific meeting), AURA Health Technologies GmbH and Siemens Healthineers.

The remaining authors declare that the research was conducted in the absence of any commercial or financial relationships that could be construed as a potential conflict of interest.

## Publisher’s Note

All claims expressed in this article are solely those of the authors and do not necessarily represent those of their affiliated organizations, or those of the publisher, the editors and the reviewers. Any product that may be evaluated in this article, or claim that may be made by its manufacturer, is not guaranteed or endorsed by the publisher.
